# Antigen-Specific Treatment Modalities in MS: The Past, the Present, and the Future

**DOI:** 10.3389/fimmu.2021.624685

**Published:** 2021-02-19

**Authors:** Judith Derdelinckx, Patrick Cras, Zwi N. Berneman, Nathalie Cools

**Affiliations:** ^1^ Laboratory of Experimental Hematology, Vaccine and Infectious Disease Institute (VaxInfectio), Faculty of Medicine and Health Sciences, University of Antwerp, Antwerp, Belgium; ^2^ Division of Neurology, Antwerp University Hospital, Edegem, Belgium; ^3^ Born Bunge Institute, Translational Neurosciences, Faculty of Medicine and Health Sciences, University of Antwerp, Antwerp, Belgium; ^4^ Center for Cell Therapy and Regenerative Medicine, Antwerp University Hospital, Edegem, Belgium

**Keywords:** multiple sclerosis, antigen-specific therapy, tolerance induction, myelin, experimental autoimmune encephalomyelitis

## Abstract

Antigen-specific therapy for multiple sclerosis may lead to a more effective therapy by induction of tolerance to a wide range of myelin-derived antigens without hampering the normal surveillance and effector function of the immune system. Numerous attempts to restore tolerance toward myelin-derived antigens have been made over the past decades, both in animal models of multiple sclerosis and in clinical trials for multiple sclerosis patients. In this review, we will give an overview of the current approaches for antigen-specific therapy that are in clinical development for multiple sclerosis as well provide an insight into the challenges for future antigen-specific treatment strategies for multiple sclerosis.

## Introduction

In autoimmune diseases, the immune system is derailed generating immunity against self. In the particular case of multiple sclerosis (MS), there are strong indications that the loss of tolerance is directed toward various myelin proteins, including myelin oligodendrocyte glycoprotein (MOG), myelin basic protein (MBP), and proteolipid protein (PLP) (1). Although the exact cause for this breach in tolerance is not yet known, it has been suggested that myelin-reactive CD4+ T lymphocytes, both of the T helper 1 (Th1) and T helper 17 (Th17) type, play a central role in the pathogenesis of MS (1–4). For instance, this is evidenced by the encephalitogenic capacity of CD4+ myelin-reactive T cells following passive transfer in experimental autoimmune encephalomyelitis (EAE) animal models (5, 6). Additionally, the fact that the strongest genetic risk factor for MS lies within the major histocompatibility complex (MHC) class II gene further underscores the importance of CD4+ T cells in MS pathogenesis (1, 7). More recently, the involvement of additional effector cells in the myelin-directed autoimmune reaction has been proposed, including myelin-reactive CD8+ T cells and B cells (4) (**Box 1**). Altogether, a complex autoimmune cascade, rather than a single culprit autoimmune response, appears to be driving MS pathogenesis, complicating the development of a targeted antigen-specific therapy for MS.

Box 1 **The immune pathogenesis of multiple sclerosis.**

**Figure 1:**
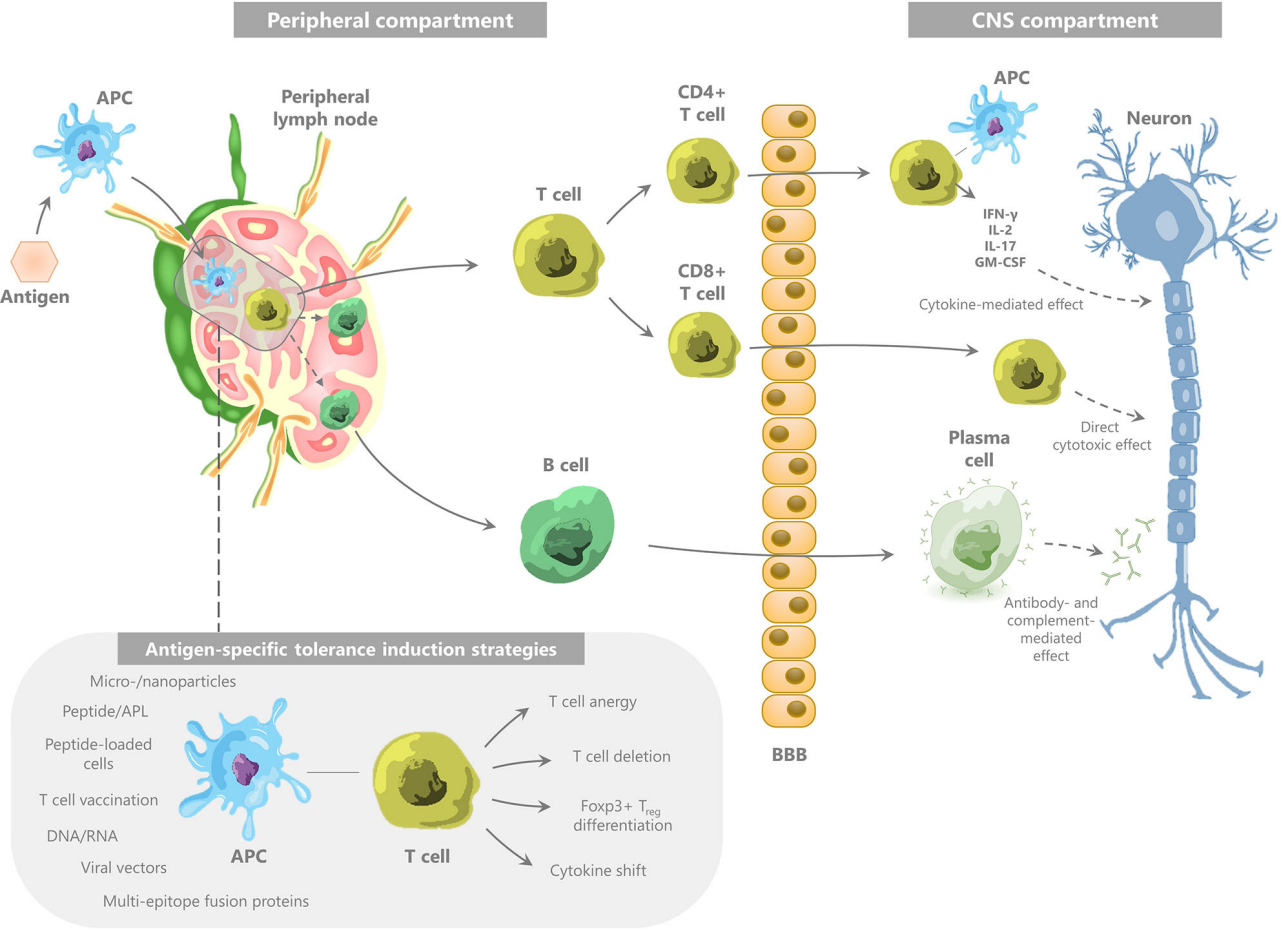
The immune pathogenesis of multiple sclerosis and the concept of myelin-specific tolerance induction.

MS is considered to be a predominantly T cell-mediated autoimmune disease (118), directed toward various myelin-derived antigens, including myelin basic protein (MBP), proteolipid protein (PLP), myelin oligodendrocyte glycoprotein (MOG), and αB-crystallin (1), that are expressed in the CNS. This autoimmunity is mostly mediated by CD4+ T cells, in particular T helper 1 (Th1) and Th17 cells (3), and involves further effectuation of an immune cascade involving CD8+ T cells, B cells, and NK cells. The exact mechanism by which these autoreactive T cells are initiated, has not been fully elucidated. As reviewed by Hemmer et al., two main hypotheses have been suggested for the immune-mediated development of demyelinating lesions (2). The first hypothesis—the so-called outside-in hypothesis—is based on peripheral activation of autoreactive CD4+ T cells recognizing CNS-derived antigens, *e.g.*, due to infection-related molecular mimicry or bystander activation (119–122). Alternatively, the inside-out hypothesis states that the initial pathogenic event takes place within the CNS, namely primary oligodendrocyte damage leading to leakage of CNS antigens to the periphery and activation of autoreactive T lymphocytes in the peripheral lymph nodes (123). However, the inside-out hypothesis is controversial, with both evidence in favor (124) and against (125) primary oligodendrocyte damage as the initiating trigger for CNS auto-immunity. Hence, the origin of the autoimmune response in MS remains a matter of debate. Nonetheless, whether the initial pathogenic event takes place in the CNS or in the periphery, one of the key elements in the immune pathogenesis of MS is the escape of autoreactive T cells from tolerance control mechanisms. This allows activated encephalitogenic CD4+ T cells to migrate across the blood-brain barrier (BBB), followed by their reactivation with autoantigens in the perivascular space (126) and their release of inflammatory mediators which activate microglia (2) (**Figure 1**). These cells will, in turn, effectuate tissue damage and produce various chemokines leading to further recruitment of effector and antigen-presenting cells (APC).

Used abbreviations: APC, antigen-presenting cell; IFN, interferon; IL, interleukin; GM-CSF, granulocyte-macrophage colony-stimulating factor; APL, altered peptide ligand; Treg, regulatory T cell.

The strong increase in knowledge regarding the pathogenesis of MS has resulted in a significant expansion of the treatment armamentarium for MS over the last years. This resulted in a wide range of disease-modifying therapeutics with varying efficacy in reducing inflammation and relapse rate. However, these therapies are accompanied by various side effects, including opportunistic infections, because of the non-disease antigen-specific mode of action resulting in a more general immune modulation or immune suppression. Hence, an ideal therapy approach for MS would aim to restore the dysregulated myelin-directed immune response without hampering the normal surveillance and effector function of the immune system (**Box 1**).

In this review, we will first give an overview of the current approaches for antigen-specific therapy that are in clinical development for MS, summarizing the results of several phase I, II and III clinical trials. In the second part of this review, we will provide an insight into the challenges for future antigen-specific treatment strategies for MS and summarize the possible solutions for these challenges that are currently being evaluated in a preclinical setting.

## Antigen-Specific Treatment in MS: Results From Clinical Trials

### Peptides and Altered Peptide Ligands

Peptide-based therapy aims to restore tolerance to specific peptides or peptide mixes by repeated administration through various routes. In parallel to hyposensitization therapy for allergy, this repeated exposure to auto-antigen induces immunological alterations, including a cytokine shift away from the autoimmune Th1/Th17 profile and induction of IL-10-secreting regulatory T cells (Treg) (8–10). Disease-related peptides can be selected by different means, including i) elution from peptide-MHC complexes (representing naturally processed peptides), ii) selection of immune dominant peptide responses by use of reactivity screening assays, or iii) prediction with computer algorithms or databases (11, 12). In addition to the use of classical peptides, altered peptide ligands (APL) can be generated by subtle modification of peptide structure, mostly by amino acid substitutions at the T cell receptor (TCR) binding site. These modifications impair T cell function following TCR-ligand interaction, which can further modulate antigen-specific T cell responses. The therapeutic potential of APL has historically been underlined by the effectiveness of glatiramer acetate, which – among other working mechanisms—acts as an APL for MBP_82–100_, causing a shift in the MBP response from a Th1 to Th2 cytokine profile [as reviewed by Schrempf et al. (13)].

Peptide- and APL-based therapy is a straightforward yet versatile approach and therefore has been the focus of many clinical trials in MS. An overview of the pivotal clinical trials focusing on peptide therapy in MS can be found in **Table 1**, which we will concisely discuss.

**Table 1:** Overview of the clinical trials using peptide therapy.

Author and year	Peptide	Trial design	Route of administration and timing	Patient population	Primary end point	Results
**Weiner et al. 1993 (** 14 **)**	Bovine myelin	Placebo-controlled phase II	Oral, daily	30 RR-MS patients	Number of severe exacerbations	Fewer severe exacerbations in treated group (6/15 versus 12/15, p=0.06)
**Goodkin et al. 2000 (** 15 **)**	MBP_84-102_ complexed to HLA-DR2	Placebo-controlled phase I	Intravenous, on day 0, 2, and 4	33 HLA-DR2+ SP-MS	Safety profile	Favorable safety profile but no effect on clinical and radiological secondary outcome measures
**Warren et al. 2006 (** 16 **)**	MBP_82-92_	Placebo-controlled phase II	Intravenous, every 6 months	32 PP-MS or SP-MS patients	EDSS progression at 24 months	No significant difference in total population In HLA-DR2- of HLA-DR4-positive subgroup: significant lower proportion of patients with sustained progression (0/10 *versus* 6/10, p=0.01)
**Freedman et al. 2011 (** 17 **)**	MBP_82-92_	Placebo-controlled phase III	Intravenous, every 6 months	528 DR2- or DR4-positive SP-MS patients 110 DR2- and DR4-negative SP-MS patients	Time to confirmed EDSS progression	No significant differences
**Yadav et al. 2012 (** 18 **)**	MOG_35-55_ complexed to HLA-DR2	Phase I	Intravenous, single injection	34 HLA-DR2+ MS-patients	Safety profile	Well tolerated up to a dose of 60 mg without increase in MS disease activity
**Walczak et al. 2013 (** 19 **)**	MBP_85-99_, MOG_35-55_ and PLP_139-155_	Placebo-controlled phase I/II	Transdermal, continuous	30 RR-MS patients	Cumulative number of active Gd+ lesions per patient per scan during the year of the study	66.5% reduction in the cumulative number of Gd-enhancing lesions compared with placebo treatment (p=0.02)
**Streeter et al. 2015 (** 20 **)**	ATX-MS-1467 (MBP_30-44_, MBP_131-145_, MBP_140-154_ and MBP_83-99_)	Phase I	Intradermal, weekly to biweekly	6 SP-MS patients	Safety profile	Safe and well-tolerated
**Chataway et al. 2018 (** 21 **)**	ATX-MS-1467	Phase Ib	Intradermal *versus* subcutaneous, weekly to biweekly	43 DRB1*15-positive RR-MS patients	Safety profile	Safe and well-tolerated. 73% decrease in new or persisting Gd-enhancing T1 lesions from baseline to week 16 (end of the treatment period) in the intradermal group versus no MRI differences in the subcutaneous group
	ATX-MS-1467	Phase IIa	Intradermal, weekly to biweekly, with a shorter titration period and longer high-dose treatment period	37 DRB1*15-positive RR-MS patients	Number of Gd+ lesions	Significant decrease in number and volume of new or persisting gadolinium-enhancing lesions, both on-treatment and post-treatment

RR-MS, relapsing-remitting multiple sclerosis; HLA, human leukocyte antigen; SP-MS, secondary-progressive multiple sclerosis; EDSS, Expanded Disability Status Scale; Gd, gadolinium; MRI, magnetic resonance imaging.

#### Peptide Therapy

Tolerance induction using peptide therapy was one of the first attempts for antigen-specific treatment for MS, with the first results on efficacy being available from phase II clinical trials at the end of the 90’s. In a phase II clinical trial, 30 relapsing remitting MS (RR-MS) patients were treated orally with bovine myelin or with control protein (14, 22). Although only 40% of patients in the group treated with myelin protein had at least one major exacerbation as compared to 80% of patients in the control group (p=0.06) (14), no conclusions regarding efficacy could be made based on these small numbers of patients (22).

Next, a placebo-controlled phase II clinical trial with intravenous administration of high doses of MBP_82–92_ was initiated by Warren et al. in 2006 (16). In this trial, 32 primary or secondary progressive MS (SP-MS) patients were treated with MBP_82-92_ intravenously every 6 months. No difference was found between treatment or placebo group in the primary endpoint, expanded disability status scale (EDSS) progression at 24 months. However, a subgroup analysis of the human leukocyte antigen (HLA)-DR2+ or DR4+ participants (20 subjects) revealed a significantly lower proportion of patients with sustained progression at 24 months in the treatment group (0/10) compared to the placebo group (6/10, p=0.01). Based on the finding that patients responded better depending on their HLA haplotype, a larger phase III clinical trial was initiated in DR2+ or DR4+ SP-MS patients (17). However, this phase III placebo-controlled trial in 612 study subjects failed to meet its primary outcome*, i.e.*, time to progression by ≥1.0 EDSS point, or ≥0.5 point if baseline EDSS was 5.5 or higher (17).

Within the context of the finding of an association between HLA haplotype and clinical effect of peptide vaccination, two phase I clinical trials have been performed using fusion products with HLA molecules. First, in 2000, a phase I dose-escalating clinical trial with intravenously administered MBP_84–102_ complexed to HLA-DR2 (AG284) in 33 HLA-DR2+ secondary MS patients was initiated, showing a favorable safety profile but no effect on clinical and radiological secondary outcome measures (15). Secondly, in 2012, a phase I dose-escalation clinical trial in 34 HLA-DR2+ MS-patients demonstrated that a fusion product of the two outer domains of HLA-DR2 with MOG_35–55_ was well tolerated up to a dose of 60 mg intravenously without increase in MS disease activity (18).

In 2013, Walczak et al. reported the results of their clinical trial with transdermal myelin peptide treatment (19). In their placebo-controlled study, 30 patients with active RR-MS were treated with a skin patch, either containing a mixture of three myelin-derived peptides (MBP_85–99_, MOG_35–55_, and PLP_139–155_) or phosphate-buffered saline (PBS). A 66.5% reduction in the cumulative number of gadolinium (Gd)-enhancing lesions compared with placebo treatment (p=0.02) was found on 3-monthly magnetic resonance imaging (MRI) scans during the first year of treatment.

In 2015, Streeter et al. reported results from a phase I clinical trial in SP-MS patients (20), which were treated with a mix of 4 MBP-derived “apitopes” or antigen processing-independent epitopes (MBP_30–44_, MBP_131–145_, MBP_140–154_, and MBP_83–99_) called ATX-MS-1467. These apitopes mimic the naturally processed T cell epitope, binding directly onto MHC class-II on immature dendritic cells (DC). This was considered to be of importance since it was previously demonstrated that attempts to induce tolerance toward a non-naturally processed epitope, *i.e.*, cryptic epitope, were not able to prevent EAE (23). Six SP-MS patients were treated with weekly to biweekly intradermal administrations of ATX-MS-1467, each receiving a dose escalation from 25 to 800 µg (20). Treatment was well-tolerated, with no major side effects. The phase Ib study, aiming to determine the optimal route of administration, showed a 73% decrease in new or persisting Gd-enhancing T1 lesions from baseline to week 16 (end of the treatment period) in the intradermal group, returning to baseline levels at week 48 (end of the off-treatment period), whereas no MRI differences could be detected in the subcutaneously treated group (24).

Immunomonitoring was performed in several of these clinical trials, demonstrating reduction in the frequency (14) and the proliferative capacity (25) of myelin-reactive T cells, a peripheral blood cytokine shift toward anti-inflammatory interleukin (IL)-10 secretion (25) and induction of myelin-specific transforming factor β (TGF-β)-secreting regulatory T cells (Treg) (26–28) following myelin peptide treatment.

In conclusion, clinical trials with peptide-based treatment have yielded both promising and disappointing results. Differences in administration route, patient population, and single-peptide- *versus* multi-peptide-based treatment may play a role in these contrasting results. At the moment, research into peptide-based therapy is continuing in MS. Currently under investigation is Neurovax^®^, a vaccine consisting of peptides derived from the T cell receptor (TCR) of pathogenic T cell clones of MS patients (29–31). Intramuscular administration of this vaccine aims to specifically modulate autoreactive T cells recognizing these peptides. Phase I clinical trials with this peptide product are currently ongoing in SP-MS and pediatric MS (NCT02200718, NCT02149706, and NCT02057159).

#### Altered Peptide Ligands

Several authors demonstrated the prevention of EAE development in rodents by administration of APL for MBP (32–37) or PLP (38–40) peptides. However, clinical translation appeared to be less unequivocal. A phase II clinical trial assessing the safety and efficacy of weekly subcutaneous administration of an APL of MBP_83–99_ (CGP77116) was halted prematurely after treatment of 8 patients because of treatment-related occurrence of MS exacerbations in 3 patients (41). Treatment with CGP77116 carried the risk of expansion of encephalitogenic MBP_83–99_-reactive T cells, as demonstrated by a strong increase in frequency of MBP_83–99_- and CGP77116-reactive T cells in peripheral blood and cerebrospinal fluid (CSF) in two of the three patients during disease exacerbation. In the same year, a second clinical trial with a different APL of MBP_83–99_ (NBI5788) was suspended after hypersensitivity reactions were observed in 9.1% of treated patients (42), even though NBI5788 was shown to be safe in a phase I study (43). Hypersensitivity was Th2-driven and arose in most patients after more than 10 administrations. Nonetheless, the volume and number of Gd-enhancing lesions 4 months after the first administration was reduced in the group of patients treated with the lowest dose of 5 mg of NBI5788 (42). Hence, induction of Th2 responses toward myelin antigens appeared to be a double-edged sword, with both beneficial and adverse effects. Similar immediate hypersensitivity reactions have been reported for glatiramer acetate, making the authors conclude that APL might be a new class of therapeutics for MS, but with the need to regulate the strength of the Th2 response (42). Nevertheless, despite the success of glatiramer acetate, no clinical trials using APL have been initiated since then, even though preclinical work on APL in EAE models still continues (44–46).

### Peptide-Loaded Cell Therapies

A phase I dose escalation clinical study was performed by Lutterotti et al., using autologous peripheral blood mononuclear cells (PBMC) coupled to 7 myelin peptides (MOG_1–20_, MOG_35–55_, MBP_13–32_, MBP_83–99_, MBP_111–129_, MBP_146–170_, and PLP_139–154_) in the presence of the chemical cross-linker 1-ethyl-3-(3-dimethylaminopropyl)-carbodiimide (EDC) (47). Seven RR-MS and 2 SP-MS patients were treated with doses ranged from 1x10^3^ to 3x10^9^ antigen-coupled PBMC, administered in one single intravenous infusion (47). No major side effects were reported. Moreover, myelin-specific T cell responses were reduced 3 months after treatment in the four patients receiving highest doses (≥1x10^9^ myelin-coupled PBMC). Two mechanisms appear to be driving tolerance induction through EDC-fixed peptide-loaded carrier cells, which are themselves deprived of their cellular function following fixation. Based on EAE data, a first mechanism consists of induction of apoptosis in myelin-reactive T cells upon antigen presentation without costimulation by the EDC-fixed carrier cells (48). In addition, a contribution of secondary cross-tolerance induction by presentation of peptides by host antigen-presenting cells (APC) following uptake and processing of the peptide-loaded carrier cells was demonstrated (48). Given the promising results of this phase I clinical trial, a phase I/II clinical trial focusing on peptide-loaded red blood cells, called ETIMSred, was initiated recently (49).

In addition to the use of fixed carrier cells, peptide-loaded cell therapy strategies can make use of viable APC as carrier cells to add a direct tolerogenic property to the peptide product. Recently, a phase Ib clinical trial was completed, demonstrating a favorable side effect profile of myelin antigen and aquaporine-4 antigen-loaded tolerance-inducing DC (tolDC) for the treatment of a mixed group of MS and neuromyelitis optica patients (50). Similarly, 2 phase I clinical trials using vitamin D3-treated tolerance-inducing DC (tolDC) loaded with a pool of myelin peptides are ongoing [NCT02618902 and NCT02903537 (51)]. These trials were initiated following promising results in a preclinical setting, with MOG_40–55-_loaded vitamin D3-treated murine tolDC showing a beneficial effect on the clinical course of EAE (52, 53).

### Myelin-Specific T Cell Vaccination

Deletion of myelin-specific T cells can be aimed for by infusion of autologous anti-myelin T cells attenuated by irradiation. By exposure of the immune system to the self-antigens carried by these attenuated T cells, a T cell response leading to deletion or downregulation of autoreactive T cells is induced (54–57). This so-called myelin-specific T cell vaccination was the subject of several open-label clinical trials, followed by a first double-blind, placebo-controlled clinical trial in 2012. In this trial, 17 relapsing progressive MS patients were treated with a mixture of autologous irradiated T cells reactive to nine different myelin-derived peptides, compared to 7 placebo-treated patients (57). In the T cell-treated group, a significant reduction in Expanded Disability Status Scale (EDSS) score 1 year after treatment could be demonstrated in comparison to an increased score in the placebo-treated group, as well as a reduced relapse rate in the T cell-vaccinated group.

### DNA Vaccination

Safety of and immune modulation by BHT-3009, a MBP-encoding DNA plasmid, was evaluated in a phase I/II clinical trial in 30 RR-MS and SP-MS patients and was demonstrated to be safe and well tolerated (58). Antigen-specific immune responses were evaluated in a subgroup of patients, demonstrating a significant decrease in myelin-specific proliferation of IFN-γ-producing CD4+ T cells at week 9 and 50 following BHT-3009 administration in all patients who displayed myelin-reactivity at baseline. Moreover, myelin-specific antibody titers were reduced in the CSF, pointing toward downregulation of myelin-specific immune responses both in the periphery and the central nervous system. Interestingly, tolerance induction was not only confined to MBP but spread to other myelin proteins, both in the CSF and in the peripheral blood.

In a larger phase II clinical trial, 289 RR-MS patients were randomized into three treatment groups comparing placebo, 0.5 mg BHT-3009, and 1.5 mg BHT-3009 (59). Administration was performed intramuscularly at week 0, 2, and 4, followed by 4-weekly administrations until week 44. Treatment with 0.5 mg of BHT-3009 led to a significant reduction in volume of enhancing lesions (51% reduction, p=0.02).

## Overcoming Challenges of Current Antigen-Specific Treatment Approaches

Although promising results have been achieved with various of the above-mentioned approaches to induce antigen-specific tolerance in MS, several challenges remain (**Table 2**). It is currently generally accepted that myelin-derived proteins are the main antigens targeted by autoreactive responses in MS (1). Nonetheless, the wide variety of MS-associated myelin-derived antigens imposes difficulties for the selection of target antigens for antigen-specific therapies. Additionally, there is a high patient-to-patient variability in myelin reactivity responses (60, 61). Moreover, these responses are often dynamic in time, characterized by loss of tolerance against additional endogenous antigens released during an inflammatory or auto-immune exacerbation. This process is also known as epitope spreading. These newly released epitopes are secondary and differ from the dominant epitopes, toward which the initial autoimmune response was targeted (62). Both intramolecular spreading*, i.e.*, development of autoreactivity against new epitopes of the initial targeted protein, and intermolecular spreading*, i.e.*, spread of autoreactivity to other myelin-derived proteins, have been described (63). Additionally, as demonstrated in the clinical trials focusing on APL, unwanted immune responses following myelin tolerization strategies—both disease exacerbations by augmentation of the targeted Th1/Th17 immune response and hypersensitivity reactions by cytokine shift to a Th2 response—remain a matter of concern. Finally, various questions remain in the light of further clinical translation of antigen-specific therapy, including optimal antigen dose and patient stratification in order to select patients likely to benefit from a specific antigen-specific treatment approach. In the following section, we will discuss different approaches to tackle these challenges.

**Table 2:** Challenges for next-generation antigen-specific treatment approaches for multiple sclerosis (MS).

****Challenge	Possible solution****	Treatment approach****
**Lack of target antigen identification, multi-epitope antigen target and epitope spreading**	Use of multiprotein and multi-epitope tolerizing strategies to induce tolerance toward a wide variety of full-length proteins	Nucleic acids, viral vectors, fusion products, peptide mixes
**Prevention of unwanted immune responses**	Targeting of antigen expression to specific cell populations	Viral vectors, fusion products
	Modification of antigen-specific T cell responses	Fusion products, nanoparticles
**Determination of optimal antigen dose for tolerance induction**	More insight into low-zone tolerance induction, optimal antigen formulation	
**Patient stratification**	More insight into parameters for selection of patients likely to benefit from antigen-specific treatment approach	

### Lack of Target Antigen Identification, Multi-Epitope Antigen Target and Epitope Spreading

#### Full-Length Protein Administration by Use of Viral Vectors or Nucleic Acids

Although still requiring knowledge of the target proteins, the use of viral vectors or nucleic acids encoding full-length myelin proteins eliminates the need for prior selection of immune-dominant epitopes, which is in line with the first attempts to induce tolerance in MS using a MBP-encoding DNA vaccine (58, 59). Indeed, following translation of full-length protein encoded by viral vectors or nucleic acids, processing by APC will ensure presentation of a wide variety of naturally processed myelin peptides in a HLA-independent manner.

Viral vector transfection is a versatile method to genetically modify several cell types, including bone marrow cells or differentiated effector cells, to constitutively express myelin proteins. Historically, the use of second-generation viral vectors, such as self-inactivating lentiviral and retroviral vectors, has reduced some of the risks related to vector-based gene therapy such as insertional mutagenesis (64). This has greatly increased the translational potential of this treatment approach. In this context, several preclinical studies demonstrated successful prevention of EAE development following treatment with bone marrow, B or T cells transfected with full-length MOG-encoding retroviral (65–70) or lentiviral (71–73) vectors, as well as with vectors encoding MBP (58, 74–76) or PLP (77). However, to our knowledge, no clinical trials in MS patients using viral vectors are yet planned.

In addition to the use of nuclide acid vaccination with DNA (58, 59), the use of mRNA is gaining interest as well, given its high clinical safety profile because of the transient expression of mRNA and its inability for host genome integration (78, 79). Although direct administration of mRNA has not been investigated in the EAE model, mRNA transfection of carrier cells to induce myelin-derived antigen presentation has been attempted. Indeed, a clinical benefit of treatment with *MOG* mRNA-electroporated tolerogenic DC (tolDC), carrying a wide spectrum of naturally processed MOG-derived epitopes, was recently demonstrated in MOG_35–55_ EAE mice (80). This protective effect was accompanied by a decrease in the MOG_35–55_-specific pro-inflammatory response in the peripheral immune system and was likely driven by suppression of central nervous system inflammation.

#### Use of Multi-Epitope Fusion Proteins

Tackling of complex multi-targeted myelin reactivity which is dynamic over time—as is the case for MS—can hypothetically be achieved by broad tolerization with a mix of myelin-derived peptides, as has already been attempted in several of the clinical trials described above, however with varying success. Ideally, antigen-specific therapy should tackle all disease-related autoreactive responses concomittantly in order to downregulate pathogenic myelin reactivity. In addition to further expanding the number of peptides in the peptide mix product, the use of artificial multi-epitope fusion proteins may be a next step forward in the field of peptide-instigated tolerance induction, since they have been demonstrated to be superior to myelin peptides mixes in preventing or downregulating EAE (81). Indeed, a globular protein product of a synthetic gene encoding different MS-associated epitopes of MBP, PLP, MOG, myelin-associated oligodendrocyte basic protein and oligodendrocyte-specific protein (designated Y-MSPc), displayed stronger capacity to induce T cell anergy, a cytokine shift, and Treg induction when compared to a similar peptide mix, resulting in more effective suppression and even reversal of EAE (81). Although the mode of action behind this stronger immunomodulatory effect by the artificial protein product remains elusive, the authors suggest multiple mechanisms, including lower degradation and clearance rate, more efficient *in vivo* uptake of Y-MSPc, different pathways of MHC-class II presentation (81) and—more recently demonstrated—induction of a specific subset of tolerogenic myeloid CD11c+CD11b+Gr1+ DC (82).

Other examples of tolerance induction in EAE using multi-epitope fusion proteins are readily available. For instance, Elliot et al. generated a fusion protein (MP4), containing full-length MBP and the three hydrophilic domains of PLP (83). Treatment of SJL/J mice with MP4 after EAE induction completely suppressed EAE development, even when EAE induction was performed using adoptive transfer of both MBP- and PLP-reactive T cells (83). Similarly, Zhong et al. demonstrated a strong preventive and therapeutic effect on EAE of a fusion protein containing encephalitogenic epitopes of MBP, MOG, and PLP (84). Interestingly, not only PLP_139–151_-induced EAE was suppressed following intraperitoneal or intravenous administration of the fusion peptide, but also EAE passively induced by T cells reactive against different myelin peptides, demonstrating the ability of the fusion protein to tackle multi-targeted myelin reactivity.  

### Prevention of Unwanted Immune Responses

#### Modification of Antigen-Specific T Cell Responses

Direct influence on the T cell response following antigen recognition can be achieved by interference with the T cell-APC interaction or by creation of a tolerogenic environment for antigen presentation, either by fusion of the antigen to tolerizing factors or by antigen presentation using micro- or nanoparticles.

T cells require three signals for full antigen-specific stimulation, *i.e.*, i) interaction of the TCR with MHC-bound antigen on the APC surface, ii) triggering of T-cell bound CD28 by costimulatory molecules CD80 and CD86, and iii) the presence of polarizing cytokines (85). Fusion of disease-specific antigens to molecules involved in this T cell-APC interaction could hypothetically result in tolerance induction by means of antigen presentation while blocking costimulatory signals. In this context, Northrup et al. generated fusion products of PLP_139–151_ with B7 pathway-targeting peptides mimicking CD28 and CTLA-4. This fusion protein interferes with the interaction with costimulatory molecules CD80 and CD86 (86). Subcutaneous administration of the fusion proteins at day 4, 7, and 10 post-EAE induction reduced EAE severity and suppressed weight loss. A cytokine shift was observed, with reduced splenocyte expression of pro-inflammatory IL-2 and GM-CSF, albeit dependent on the particular peptide that was used (86). To the same extent, bifunctional peptide inhibitors (BPI) have been developed to modify T cell responses. BPI consist of antigenic peptides conjugated to adhesion peptides, binding respectively to MHC and costimulatory or adhesion molecules on APC. Binding of a BPI hampers translocation and segregation of the MHC/TCR and costimulatory molecule complexes, preventing the formation of immunological synapse and subsequent T cell activation (87, 88). For instance, Kobayashi et al. demonstrated that linking of PLP_139–151_ to CD11a_237–246_, an intercellular adhesion molecule (ICAM)-1-binding peptide, suppresses PLP-induced EAE severity and incidence. The linked peptide was more effective when compared to a mixture of PLP_139–151_ and CD11a_237–246_ peptides (87). To broaden the antigen-specific immune modulation, thereby tackling epitope spreading, Badawi et al. generated a bivalent BPI consisting of both MOG_38–50_ and PLP_139–151_ bound to an adhesion molecule. In doing so, both MOG_38–50_- and PLP_139–151_-induced EAE was suppressed (88). In the same animal models, the bivalent BPI was superior to MOG_38–50_-BPI and PLP_139–151_-BPI alone for the induction of tolerance (88).

Fusion of myelin epitopes to cytokines or other active compounds by covalent binding can be used to skew the antigen-specific response toward a more tolerogenic profile. Binding of the fused cytokine to receptors on APC leads to specific targeting of the neuroantigen to these APC and enhanced antigen presentation (89). Neuroantigen-fusion proteins with granulocyte macrophage-colony stimulating factor (GM-CSF), being a major cytokine involved in development and differentiation of myeloid APC (90), displayed a more than 1000-fold increase in antigen targeting to APC compared to neuroantigen alone (91). Accordingly, subcutaneous administration of GM-CSF-neuroantigen fusion proteins has shown to be effective in the prevention and treatment of MOG_35–55_- (92, 93), PLP_139–151_- (92, 93) and MBP_69–87_ (89, 91)-induced EAE. Similarly, fusion proteins of myelin proteins with IFN-β (89, 94), IL-16 (89, 95), IL-13 (89, 95), IL-10 (95), IL-2 (89, 95, 96), IL-4 (89), and IL-1RA (89, 95) have been tested in Lewis rat or SJL mice EAE models. Of these, IFN-β and IL-16 gave the highest tolerogenic capacity, however still less effective than GM-CSF (89). In all settings, cytokine-neuroantigen fusion proteins were superior in terms of inhibitory capacity over neuroantigen alone (89, 95), which underlines the benefit of antigen targeting to APC.

#### Targeting of Antigen Expression to Specific Cell Populations

Targeting of myelin expression to specific cells can enhance tolerance induction and reduce off-target effects by specifically guiding the myelin presentation to possibly tolerogenic environments. For instance, following viral transfection, ubiquitous myelin expression can be prevented by targeting specific cell lineages by using vectors in which expression is under the transcriptional control of specific cell-type promotors. Cell lineages of interest include DC (70–72), as major APC controlling the balance between tolerance and immunity, and hepatocytes (74, 97), being part of the tolerogenic environment of the liver. Several viral vector-based cell-targeting treatment approaches have been attempted in the EAE model, which are described in **Table 3**.

**Table 3:** Preclinical evaluation of viral vector transfection, targeting specific cell types, for tolerance Figinduction in EAE.

Author and year	Protein or peptide encoded	Administration approach	Cell type targeted	Animal model	Clinical setting	Results and mode of action
**de Andrade Pereira et al. 2013 (** 71 **)**	Full-length mouse MOG in SIN lentiviral vector	IV transfer of transduced HSC into irradiated C57BL/6 mice	DC by use of DC-STAMP promotor	MOG_35-55_-induced EAE in C57BL/6 mice	Preventive (EAE induction 8 weeks after HSC transfer)	Full protection by deletion of MOG-specific T cells and generation of Treg
**de Andrade Pereira et al. 2015 (** 72 **)**	Full-length mouse MOG in SIN lentiviral vector	Transfer of transduced BM cells into irradiated C57BL/6 mice	DC by use of DC-STAMP promotor	Passive transfer of 2D2 T cells into C57BL/6 mice	Preventive (transfer 8 weeks before passive EAE induction)	Full protection by induction of unresponsiveness of preactivated MOG-specific CD4+ 2D2 T cells to MOG and acquisition of an anergic or regulatory phenotype by transferred cells
**Eixarch et al. 2009 (** 98 **)**	MOG_40-55_ into Ii molecule in retroviral vector	IV transfer of transduced BM cells into C57BL/6, either partially myeloablated or not myeloablated	MHC class II targeting by replacement of the CLIP-encoding region of the murine Ii molecule by MOG_40-55_	MOG_40-55_-induced EAE in C57BL/6 mice	Preventive (transfer 21 days before EAE induction) or therapeutic (transfer 15–17 days after EAE induction)	Protection from EAE development in preventive setting, amelioration of clinical score in therapeutic setting, with increase in IL-5 and IL-10 secretion by splenocytes, pointing towards involvement of Treg
**Fransson et al. 2012 (** 73 **)**	CARαMOG-FoxP3 construct in lentiviral vector	Intranasal transfer of transduced T cells into C57BL/6 mice	CD4+ T cells by direct transfection, Foxp3 driving Treg differentiation	MOG_35-55_-induced EAE in C57BL/6 mice	Therapeutic (transfer approximately at day 15 after EAE induction, at clinical score of 3)	Reduction of disease symptoms and protection from EAE rechallenge, with reduction of mRNA expression of IFN-γ and IL-12 in the CNS
**Keeler et al. 2017 (** 97 **)**	Full-length MOG in adenovirus-associated vector	IV administration of vector into C57BL/6 mice	Hepatocytes by use of hepatocyte-specific promoter	MOG_35-55_-induced EAE in C57BL/6 mice	Preventive (transfer 2 weeks before EAE induction) and therapeutic (at different clinical scores)	Protection from EAE development in preventive setting, reversal of mild-to-moderate clinical symptoms in therapeutic setting, reversal of severe clinical symptoms in combination with rapamycine in therapeutic setting, by induction of MOG-specific Treg
**Ko et al. 2011 (** 70 **)**	Full-length mouse MOG in SIN retroviral vector	IV transfer of transduced BM cells into irradiated C57BL/6 mice	DC by use of CD11c promotor	MOG_35-55_-induced EAE in C57BL/6 mice	Preventive (transfer 8–9 weeks before EAE induction)	Delay in EAE development, but no protection, no mechanistical analyses were performed
**Luth et al. 2008 (** 74 **)**	MBP splice variant in type 5 adenoviral vector	IV administration of vector into FVB mice	Hepatocytes by use of type 5 adenoviral vector	MBP_1-9_-induced EAE in FVB mice	Preventive (transfer 2 weeks before EAE induction)	Protection from EAE development, by induction of MBP-specific Treg by TGF-β-driven conversion from conventional CD4+CD25− T cells

IV, intravenous; HSC, hematopoietic stem cell; DC, dendritic cell; DC-STAMP, dendritic cell-specific transmembrane protein; Treg, regulatory T cell; SIN, self-inactivating; BM, bone marrow; MHC, major histocompatibility complex; IFN, interferon; IL, interleukin; TGF, transforming growth factor.

Additionally, fusion proteins can be used for direct targeting. Ring et al. generated a fusion protein of MOG_35–55_ and single-chain fragment variables (scFv) specific for DEC205, which is a receptor almost exclusively expressed by DC (99). Injection of this fusion protein was shown to be beneficial for both EAE development and progression when mice were treated before (preventively) or after (therapeutically) disease induction, respectively (99). MOG_35–55_ expression was targeted to DC, which led to significantly reduced levels of TGF-β secretion by DC and increased numbers of IL-10-producing Treg in the spleen (99). Similarly, a fusion product of MOG_35–55_ and anti-Siglec-H antibodies targeted MOG expression to plasmacytoid DC (pDC) and delayed or decreased clinical signs of EAE when administered in a preventive setting or therapeutic setting, respectively (100).

#### Micro- and Nanoparticle-Based Systems

Following the success of antigen-coupled cell therapy, micro- and nanoparticles were developed as a delivery vehicle for autoantigens, circumventing the need for autologous blood cells, thereby enhancing clinical translation (101). Micro- and nanoparticles can be used as antigen-delivering vehicles that prevent unwanted immune responses using the strategies mentioned above. Indeed, as reviewed by Kishimoto et al., three strategies can be used for tolerance induction using nanoparticles (102). First of all, nanoparticles can make use of natural tolerance processes, such as antigen presentation without costimulation, oral tolerance, or delivery to the tolerogenic liver environment. For instance, Carambia et al. demonstrated a clinical improvement in EAE mice following a single dose of autoantigen-loaded nanoparticles, specifically targeting to liver sinusoidal endothelial cells, associated with a significant higher frequency of Treg in the spleen of nanoparticle-treated mice compared to vehicle-treated mice (103). Secondly, nanoparticles can be used to specifically target tolerogenic receptors. As an example, a nanoparticle containing MOG_35–55_ and a plasmid containing the murine B and T lymphocyte attenuator (BTLA) was created by Yuan et al. (104). Following transfection of DC with this plasmid and subsequent administration of these transfected DC prior to induction of MOG_35–55_ EAE, EAE development could be prevented and was accompanied by an increased frequency of Treg (104). A final approach is to use nanoparticles to co-administer autoantigens together with tolerogenic pharmacological agents, which has been used in the context of EAE in combination with rapamycine (105, 106) and dexamethasone (107). In conclusion, micro- and nanoparticles have been shown to be a versatile treatment modality in preclinical setting, yet no clinical trials are ongoing currently.

### Determination of Optimal Antigen Dose

Auto-antigen dose is often extrapolated from dosing from animal models or determined by safety studies, in which the maximal tolerable dose is considered to be the dose of choice. However, the concept of low-zone tolerance, in which low antigen doses are superior in inducing tolerance compared to high doses, has already been known for several decades (108–110). Indeed, also in the context of MS, Garren et al. demonstrated in their phase II clinical trial with the DNA vaccine BHT-3009 that the 0.5 mg group was significantly superior in inducing tolerance compared to the 1.5 mg group, as demonstrated by MRI measures and *in vitro* PLP reactivity (59). Similarly, as demonstrated by Kappos et al. in their phase II clinical trial using an APL derived from MBP_83–99_, a significant decrease in the volume and number of Gd-enhancing lesions could only be detected in the patient group treated with the lowest dose (42). On the other hand, high-zone tolerance has been demonstrated for tolerance induction in other autoimmune diseases, including hemophilia (111), leaving the efficacy of low-zone *versus* high-zone tolerance to be determined for every tolerance-inducing strategy on an individual base. In conclusion, determination of optimal dosing should be based on both tolerability and efficacy.

In addition, optimization of the antigen product formulation to ensure sufficient antigen delivery is warranted for each particular route of administration, since delivery of an appropriate dose of the auto-antigen to the site of interest is of crucial importance for the effective induction of tolerance. For instance, upon oral administration of peptides, passage of low-dose antigen through the gut-associated lymphoid tissue (GALT) induces antigen-specific regulatory T cells (Treg) in the Peyer’s patches (22). However, suppression of ongoing autoimmune reactions, as needed in a therapeutic setting, requires large amounts of oral antigen intake, limiting the clinical applicability of this technique (112, 113). Therefore, generation of fusion proteins with higher efficacy should be aimed for, in which the antigen is either directly targeted to the GALT, e.g., by fusion to cholera toxin subunits (114, 115), or in which higher presentation efficacy can be achieved by fusion to cell membrane-associated proteins (24). Similarly, repeated nasal administration of a fusion protein consisting of cholera toxin subunit B and PLP_139–151_ hampered full EAE development (114). Hence, also for the nasal route of administration, formulation of the auto-antigen should be optimized.

### Patient Stratification

Selection of patients likely to benefit from a particular antigen-specific therapy would aid in the development of patient-tailored therapies. Based on subgroup analyses, the HLA-DR haplotype has been demonstrated to be a parameter of importance in the immunological and clinical response to the induction of myelin-specific tolerance. This is not surprising, giving the role of APC-bound HLA-DR in the antigen presentation to CD4+ T cells. The importance of the HLA-DR haploptype is especially the case for antigen-specific tolerance induction strategies using peptides, given that some myelin peptides are HLA-restricted (116, 117), meaning that they are preferentially presented by specific HLA-molecules. However, clinical trials using HLA-DR haplotype as an inclusion parameter have yielded conflicting results. This is most likely due to confounding by other parameters, which should be taken into account for patient selection as well. This includes among others the presence of pre-treatment reactivity toward the epitopes contained in the antigen-specific therapy. Although of major importance in order to be able to assess antigen-specific immune modulation following treatment, pre-treatment myelin-specific reactivity has not been consistently determined in previously conducted clinical trials, limiting the comparative evaluation of the treatment effect on an immunological level.

## Conclusion

Numerous attempts to restore tolerance toward myelin-derived antigens have been made over the past decades, both in animal models of MS and in clinical trials for MS patients. Many of these treatment approaches have shown to be safe and well-tolerated in phase I/II clinical trials, although results regarding efficacy have appeared to be less unequivocal. Given the complexity of the myelin response to be down-regulated, patient selection in terms of HLA haplotype, myelin reactivity, and previous treatment profile is warranted. This would allow efficacy analysis in a more homogeneous patient population and may guide us in the selection of patients who may potentially benefit from a particular treatment. Indeed, a one-treatment-fits-all approach is unlikely to be successful in the field of antigen-specific therapy for MS, underlying the need for more insight into parameters for patient stratification.

Additionally, current preclinical research is providing new approaches to tackle some of the challenges faced by the currently used approaches, including epitope spreading and unwanted immune responses following myelin tolerization attempts. These new findings should altogether allow to modify currently used antigen-specific approaches with the aim to enhance their clinical efficacy.

In conclusion, several decades of research into antigen-specific therapy for MS has yielded promising results and findings from currently ongoing preclinical work may add to the efficacy of this type of treatment. Ultimately, antigen-specific therapy for MS may lead to a more effective therapy for MS by induction of tolerance to a wide range of myelin-derived antigens without hampering the normal surveillance and effector function of the immune system.

## Author Contributions

Conceptualization, JD and NC. Writing—original draft, JD. Writing—review and editing, JD, PC, ZB, and NC. All authors contributed to the article and approved the submitted version.

## Conflict of Interest

The authors declare that the research was conducted in the absence of any commercial or financial relationships that could be construed as a potential conflict of interest.
